# Circuit and Noise Analysis of Odorant Gas Sensors in an E-Nose

**Published:** 2005-02-28

**Authors:** Fengchun Tian, Simon X. Yang, Kevin Dong

**Affiliations:** 1 College of Communication Engineering, Chongqing University, Chongqing, P.R. China 400044; 2 School of Engineering, University of Guelph, Guelph, ON, Canada, N1G 2W1

**Keywords:** gas sensor, circuit, noise, power spectrum, probability distribution function

## Abstract

In this paper, the relationship between typical circuit structures of gas sensor circuits and their output noise is analyzed. By using averaged segmenting periodical graph and improved histogram estimation methods, we estimated their noise power spectra and optimal probability distribution functions (*pdf*). The results were confirmed through experiment studies.

## Introduction

Electronic noses (e-noses) are more and more widely used in environmental monitoring, food production and medicine such as odour evaluation [1-4]. The response of all sensors in the e-nose together constitutes a unique profile that gives the “fingerprint” of odour. The noises from the sensor array comprised by several odorant gas sensors may result in inaccurate cluster analysis of the tested material [5]. In our experiments, it is observed that the noise of gas sensors cannot be ignored. In the worst case, the noise magnitude could become up to 20% of the signal magnitude of some sensors. The gas sensors of the e-nose have the following features:

(1) The sensors in the array interfere with each other.
(2) Different types of sensors are used, some of which have large heating current and power dissipation.
(3) Some sensors require amplifiers with extremely high input impedance (e.g., higher than 10^11^ Ohm) that makes them susceptible to interference.
(4) Some sensors have large dynamic current that produces electromagnetic disturbances to the output of other sensors in the same array.

The drawback of the gas sensors lies in:

(1) The sensors are very sensitive to temperature and humidity.
(2) The baseline of sensors shifts with time.
(3) Large noise exists in sensor output.

**Figure 1.**
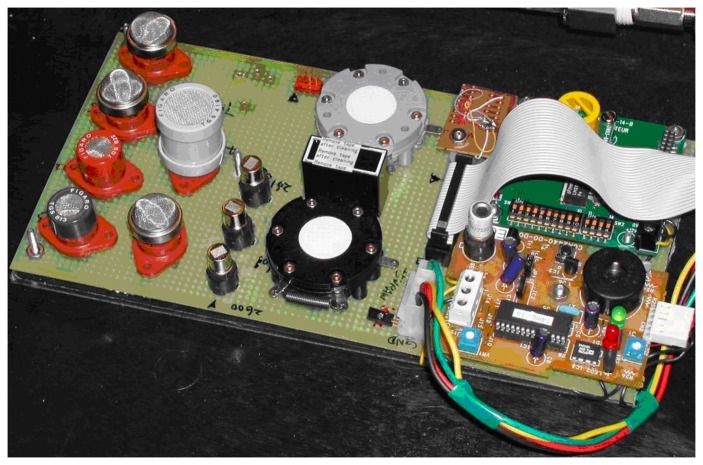
Photo of the electronic nose developed in our research laboratory.

This paper is to study the noise features of several typical gas sensors used in the e-nose developed in our research laboratory (shown in Figure 1), including their probability distribution functions (*pdf*) and power spectrum estimation, which are essential in noise cancelling [6-8] and odour analyzing by noise power spectrum [9].

## Typical gas sensors and their noise

### Resistive gas sensors

**Figure 2.**
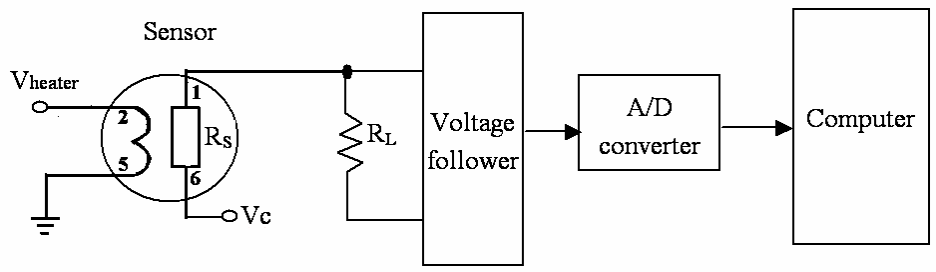
Resistive gas sensor and its interface circuit diagram.

The resistive gas sensors we studied are of MOS (Metal-Oxide Semiconductor) such as tin dioxide. Figure 2 shows a typical sensor circuit and its interface diagram. Pins 2 and 5 are connected to the heater of the sensor, and the resistance between Pins 1 and 6 is designated as R_s_. With pure air R_s_ is high. With the presence of detectable gases, R_s_ changes with the variation of gas concentration. Since V_C_ is a fixed voltage, by measuring the voltage on the resistor R_L_, the change on R_s_ as well as the gas concentration can be calculated.

This type of sensor features largely in its heater power dissipation ranging from several hundreds of milliwatt to 1 watt. The working temperature is as high as 400 °C. This will result in higher resistor thermo-noise. Besides, there exist typical semiconductor noises such as Schottky noise, flicker noise etc. As the output signal of the sensor is large enough, the interface circuit uses only a voltage follower as a buffer between the sensor output and the A/D converter, which makes the system less sensitive to external interferences.

### Gas sensors with electromotive output

**Figure 3.**
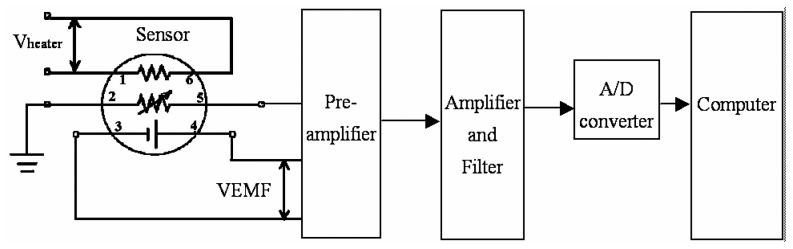
Electromotive gas sensor and its interface circuit diagram.

Figure 3 shows the circuit of a gas sensor with electromotive output and its interface diagram. There is a heater between Pins 1 and 6. A thermometer for temperature compensation is between Pins 2 and 5. The electromotive VEMF between Pins 3 and 4 reflects the concentration of detectable gas. Lower capability of payload is the main feature of this type of sensor. It requires a pre-amplifier with extremely high input impedance at a value over 10^11^ Ohm. As a result, it has the disadvantage of being vulnerable to external electromagnetic interferences in addition to the noise of the resistive gas sensor.

### Sensors with dynamic heating

**Figure 4.**
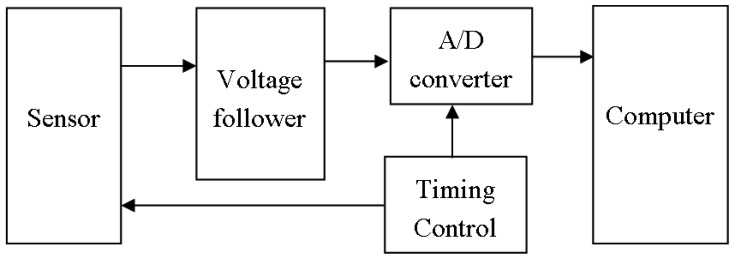
Schematic diagram of an odorant gas sensor with dynamic heating and its interface.

**Figure 5.**
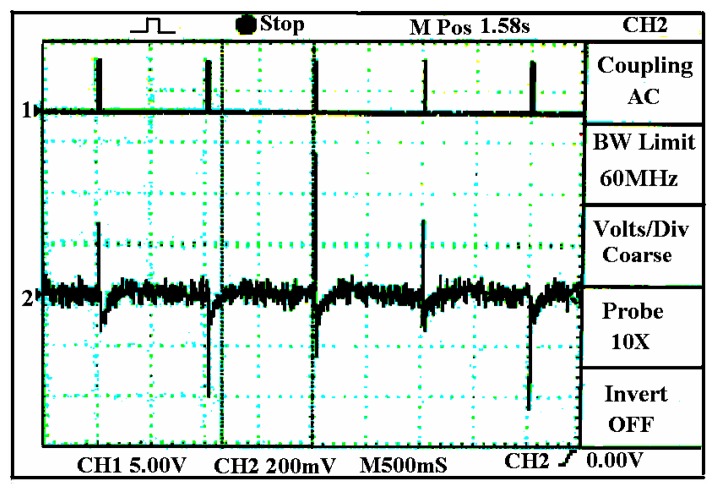
Interference on an odorant gas sensor with electromotive output.

The above two types of sensors have a fixed voltage for their heaters, the current of which is constant, whereas the current of the sensor in Figure 4 is dynamic. The difference between this type of gas sensors and the one in Figure 2 lies in the voltage on the sensor heater. Here, the voltage on the heater is a pulse signal that results in a large dynamic current as well as electromagnetic interference to other sensors in the sensor array of the e-nose. The timing control circuit is used to control the voltage applied to the sensor heater and the sampling epoch. Figure 5 is an oscilloscope-recorded waveform about the interference before any noise cancelling measure is taken, where the upper channel is the voltage waveform of heater in Figure 4, which also indicates the current on the heater. The lower channel in Figure 5 is the output waveform of another electromotive gas sensor in pure air that should be a straight line without the interference.

### Gas sensors with current output

**Figure 6.**
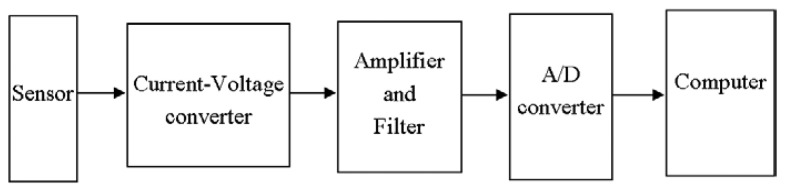
Gas sensor with current output and its interface circuit diagram.

Figure 6 shows a typical electrochemical gas sensor and its interface diagram. The working current and voltage of the circuit are low and do not produce interference to other sensor's output. Since the sensor's output current is extremely weak (only several micro amps), it requires the gain of the amplifying circuit be up to several thousands. Thus, the whole circuit is more sensitive to external disturbances from other sensors than that of sensors with a lower amplifier gain. The noise is also amplified by several thousand times and it has stricter requirement on the equivalent input noise of the amplifier.

## Probability distribution function (*pdf*) of noise

The histogram method, which has been proved to be an unbiased estimation for a random variable, is used to estimate the *pdf* of noise. The estimation error decreases at a rate of *N*^−2 / 3^ as a function of the total number *N* of the samples [10].

Let *X*_1_, *X*_2_, …, *X_N_* be independent and identically distributed on [0,1] with *N* the total number of samples. Let *m* be an integer and *h* = 1/*m*. Define the bins as

(1) $${\text{B}}_{1}=\left[0,h\right),{\text{B}}_{2}=\left[h,2h\right),\ldots B_{m}=\left[\left(m-1\right)h,1\right]$$

Let *p̂_j_* =*v_j_/N* and let *v_j_* be the number of observations in *B_j_*. Then the histogram estimation of probability distribution function *f* (*x*) is given as:

(2) $${\hat{f}}_{n}(x)=\{\begin{array}{cc} {\hat{p}}_{1}/h & x\in B_{1}\\ {\hat{p}}_{2}/h & x\in B_{2}\\ \ldots & \\ {\hat{p}}_{m}/h & x\in B_{m} \end{array}$$

The following two questions should be answered when the histogram estimation method is used:

1) How to measure the error between *f̂_n_* (*x*) and the real probability distribution function *f* (*x*) ?, and
2) What is the optimal *h* that makes the approximation of *f̂_n_* (*x*) to *f* (*x*) the best?

For the first question, we use the risk function or mean integrated squared error *R* (*f*, *f̂*; between the two functions to measure the error between *f̂_n_* (*x*) and the real probability distribution function *f* (*x*). The function *R* (*f*, *f̂*; is given by:

(3) $$R(f,\hat{f})=E\left\{\int {\left(f(x)-\hat{f}(x)\right)}^{2}dx\right\}$$

where **E**{·} is to calculate the expected value (mean). For the second question, it is obvious that the smaller the *R* (*f*, *f̂*;, the more closer will be *f̂_n_* (*x*) and *f* (*x*) (with the exception of only some point sets with zero measure). When *R* (*f*, *f̂*) reaches its minimum, the best approximation of f̂*_n_* (*x*) to *f*(*x*) is obtained. Since *f*(*x*) is unknown, *R*(*f*,*f̂*; cannot be calculated directly, but its minimum can be reached by making *Ĵ*(*h*) the minimum [10]. The function *Ĵ*(*h*) is given by:

(4) $$\hat{J}(h)=\frac{2}{(n-1)h}+\frac{n+1}{n-1}\sum_{j}^{m}p_{j}^{2}$$

The optimal *h* that results in the minimum value of *Ĵ*(*h*) can be obtained by increasing the value of *m* from 1. Here, the corresponding formula in [10] is improved by changing its minus sign between the first and the second item into a plus sign according to our experiments. Otherwise, it cannot converge.

## Power spectrum estimation of noise

The periodical graph and averaged segmenting periodical graph methods were used to obtain the power spectrum estimation of noise [11]. For a periodical graph, the power spectrum estimation is given as:

(5) $$\begin{array}{cc} S(k)=\frac{1}{N}{\left|\hat{X}(k)\right|}^{2} & k=0,1,\ldots N-1 \end{array}$$

where |*X̂* (*k*)| is the modulus of the Discrete Fourier Transformation (DFT) of *X*_1_, *X*_2_, …, *X_N_*, and *N* is the total number of data sets.

To highlight the feature of noise, the mean value of data is removed before the DFT. Otherwise, the d.c. component (zero frequency) will be too large to show other components. The variance of periodical graph is bigger than that of the averaged segmenting periodical graph, but it is useful in observing the baseline shifting of sensors (it corresponds to the lower frequency components near zero frequency).

The averaged segmenting periodical graph is obtained by segmenting the data X_1_, X_2_, …, X*_N_* into *K* small non-overlapping sections. The length of each small section is *M* with *KM* = *N*. First, the power spectrum is estimated for each small section (also remove the mean of the small section before the DFT). Then, the power spectrum estimation will be the average of the *K* small sections. The disadvantage of this method lies in its losing of information on sensor's baseline shifting that is much smaller during the small section than that of the whole section. The advantage is its asymptotic consistent estimation of real power spectrum. With the increase of *M*, the variance of the averaged segmenting periodical graph approaches zero. By this manner, the power spectrum estimation of the small section *j* is given as:

(6) $$\begin{array}{cc} S_{j}(k)=\frac{1}{M}{\left|\hat{X}(k)\right|}^{2}, & k=0,1,\ldots ,M-1 \end{array}$$

where *X̂* (*k*) is the DFT of *X*_1_, *X*_2_, …, *X_M_*. The total power spectrum estimation is given as:

(7) $$\begin{array}{cc} S(k)=\frac{1}{K}\overset{K}{\underset{j=1}{\text{∑}}}S_{j}(k), & k=0,1,\ldots ,M-1 \end{array}$$

## Experiment results

Four types of sensors are used in the test: (1) resistive gas sensors (such as TGS813); (2) gas sensors with electromotive output (such as TGS4160); (3) gas sensors with dynamic heating (such as TGS2442); (4) gas sensors with current output (such as 3ETO). The first three types of sensors are MOS sensors produced by Figaro Ltd., while the forth ones are electrochemical gas sensors produced by City Technology Ltd.

**Figure 7.**
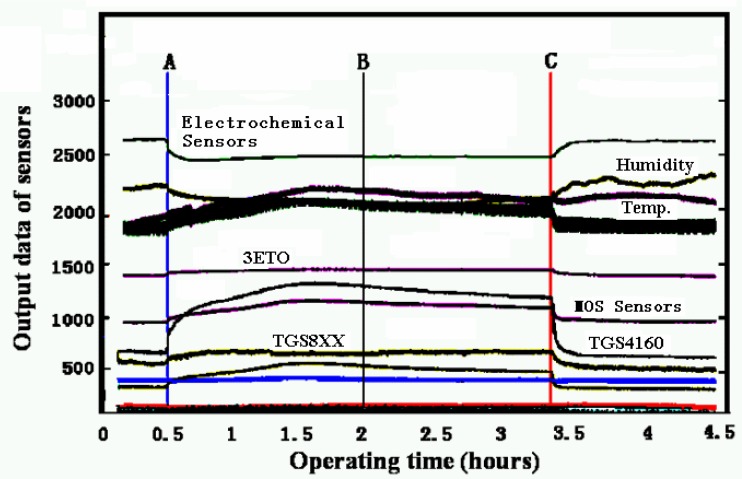
Power supply and response of the16 sensors in e-nose.

Figure 7 depicts the response of the above sixteen sensors, each of which is sampled by a 12-bit A/D converter at 6.4 Hz sampling frequency. A charcoal filter (which results in pure air input) was used from epoch A. Since no detectable gas appears, the output of sensors is noise only. A pump was used to intake the pure air into a chamber within which the sensor array lies. After epoch C, the chamber was opened which resulted in the temperature dropping. To get the feature of noise, the stabilized data (from epoch B to C) was used to compute the power spectrum and *pdf* estimation. The number of data sets for each sensor used in calculation is 30,000. In averaged segmenting periodical graph method, the data were segmented into *K*=6 small sections with the length of each one as *M*=5,000. In depicting the *pdf* curve, to highlight the non-zero part, without the loss of generality, we normalized the sensor's output data into [0,1], i.e., let the maximum and minimum of sensor output data be *X*max and *X*min, respectively. Then the normalized data will be given as:

(8) X=(X−Xmin)/(Xmax−Xmin)

Our experiments show that the sensors can be categorized into three types according to their noise power spectrum and *pdf* estimation (see Table 1). Here, a white noise means that its power spectrum magnitude keeps almost constant in the whole frequency range except the d.c. and its nearby component, while a coloured noise contains considerable low frequency components besides that of the white noise.

**Figure 8.**
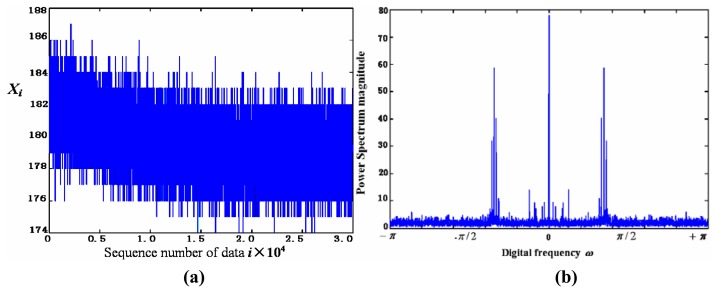
The noise (a) and its power spectrum estimation (b) of the first type of sensors in Table 1.

**Figure 9.**
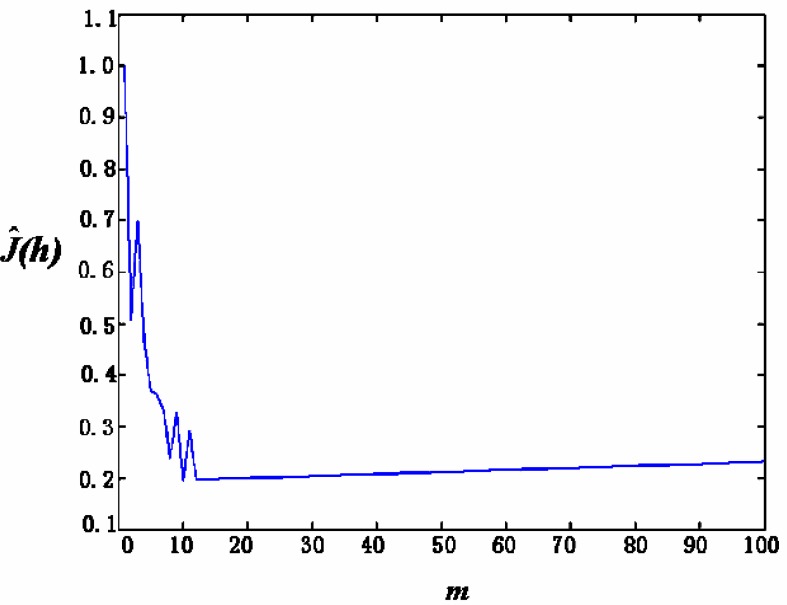
*Ĵ*(*h*) as a function of *m*.

**Figure 10.**
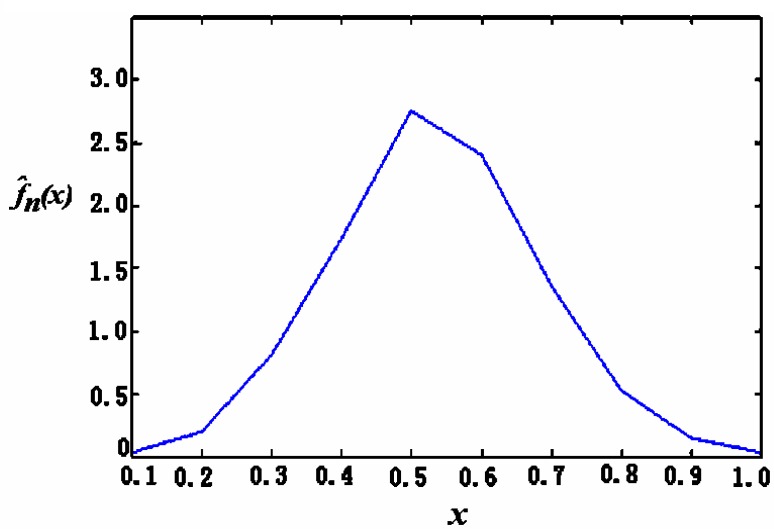
Histogram under the optimal value of *h*.

**Figure 11.**
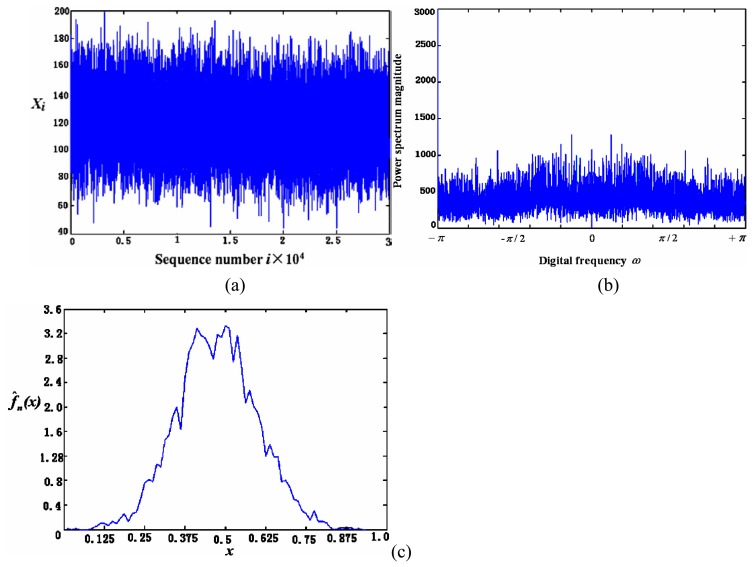
The noise (a), its power spectrum (b) and *pdf* estimation (c) of the second type of sensors in Table 1.

**Figure 12.**
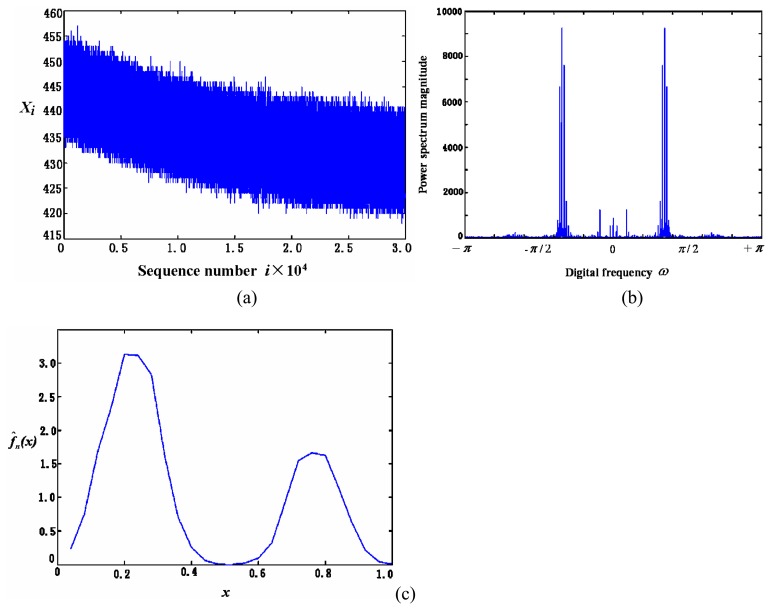
The noise (a), its power spectrum (b) and *pdf* estimation (c) of the third type of sensors in Table 1.

**Figure 13.**
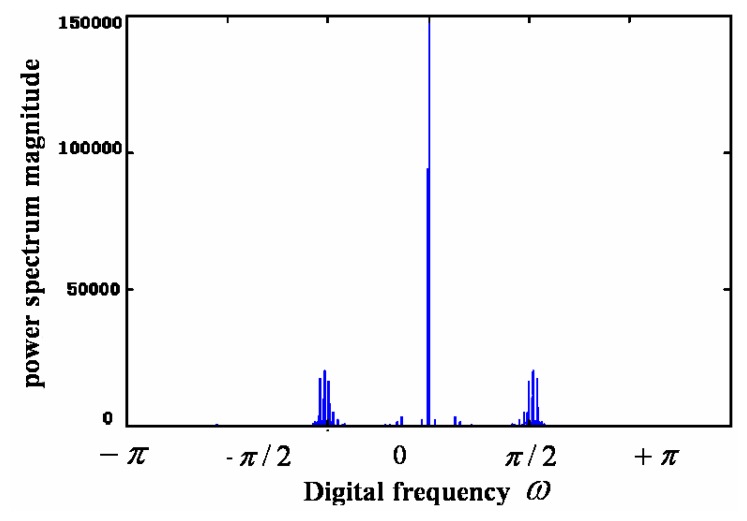
Power spectrum by periodical graph.

(1) For the case of coloured noise with a single peak *pdf*, Figures 8(a) and 8(b) show the time-domain curve and the power-spectrum estimation curve, respectively. It shows that the power spectrum of noise mainly consists of two parts: one is the almost constant-magnitude part filling the whole frequency band, and the other is composed of some lower frequency components that may be caused by some inherent feature of the sensor and its circuit. Some of them have the same frequency as signal; it cannot be filtered out by just a simple low-pass filter.
  Figure 9 shows the curve of *Ĵ*(*h*) from Equation (4) with *h* = 1/*m*. It can be seen that when *m* = 10, i.e., *h* = 0.1, *Ĵ*(*h*) reaches its minimum that makes the risk function *R* (*f*, *f̂*; minimum. The *h* value at *h* = 0.1 makes the estimation of *pdf* to be optimal. Figure 10 gives the *pdf* estimation under this *h* value. It is close to a Gaussian distribution.
(2) In the case of white noise with single peak, the noise in time domain and its power-spectrum estimation curve are shown in Figures 11(a) and 11(b), respectively. The *pdf* estimation is shown in Figure 11(c) with the optimal value at *h* = 1/75. It is similar to a Gaussian distribution. The envelop of the noise power spectrum is almost constant in the whole band. Thus, it is reasonable to consider this type of noise as normal white noise. For the sensors 3SH (current output) and TGS4160 (electromotive), their output signals are too weak and the gain of amplifier has to be large enough (about 6,000), or the input impedance is too high (more than 10^11^ Ohm). The noise energy of these sensors is much bigger than that of the first type in Table 1, which implies that the noise in this type of sensor cannot be ignored.

(3) For the coloured noise with double peak *pdf*, Figures 12(a) and 12(b) show the noise in time domain and its power spectrum estimation of sensor, respectively. Figure 12(c) is the estimation of *pdf* with the optimal value at *h* = 1/29. In comparison to the first type of sensors in Table 1, both have similar coloured noise power spectrum and many of their low frequency components have the same location in the frequency domain. This implies that the inherent features of the sensors are similar, as they are produced by the same manufacturer. The only obvious difference here is that its *pdf* has double peaks, while the first type has only one. The double peaks in its *pdf* imply the high appearance frequency of these two types of noise magnitude.

(4) For some sensors such as TGS830, TGS2600, the remarkable difference exists in their zero frequency component of noise power spectrum between the periodical graphs and averaged segmenting periodical graph methods. This can be observed when comparing Figure 12(b) and Figure 13. No peak appears in the nearby zero frequency of the averaged segmenting periodical graph in Figure 12(b), which is opposite to its counterpart in Figure 13. It is due to the fact that the sensor baseline shifts slowly within the whole time period (the total 30,000 data sets), although in both methods the mean is removed. Whereas in each small section (5,000 data sets) the baseline changes little and results in almost no nearby zero frequency component. That indicates, of all sensors tested, the three types of sensors can work more stably and have much smaller baseline shift than other sensors.

## Conclusions

The sensor and its interface circuit of four types of odorant gas sensors of an electronic nose are investigated and their noise features are analyzed. Several points are worth to be mentioned:

1. Since the sensors only work with its corresponding circuits as a whole, the sensor noise here means the whole noise from the sensor and its amplifying circuit. The noise is related to the art of manufacture, the input impedance of amplifier and its gain. In our experiment, the optimal operating circuits and devices recommended by the manufacturers are adopted. So it is reasonable to believe those e-noses which use the same type of sensors have similar circuit structure and noise feature as we give here.
2. The noise in the sensors of the e-nose can be categorized into coloured or white noise according to its power spectrum. The coloured noise can be regarded as the white noise plus some stronger low frequency components. The *pdf* of noise is of either single peak or double peaks.
3. The noise of resistive gas sensor is coloured one.
4. The white noise lies in either electromotive sensor or sensor with current output, which is due to either the weak signal or the high input impedance.
5. To estimate the *pdf* of noise, we have used the optimal width (*h*) for bins in histogram that results in the closest estimation to the real *pdf* in the sense of smallest integrated mean squared error. The averaged segmenting periodical graph estimation of noise power spectrum is an asymptotic consistent estimator of the real power spectrum. The error of estimation is expected to be small enough considering the huge number of data sets.
6. The baseline shift of sensor can be observed through comparing the periodical graph and the averaged segmenting periodical graph of noise. Of all the sensors tested, the three sensors TGS830, TGS2600, TGS2602 can work more stably and have smaller baseline shift than others. They almost have no baseline shift in each small section of 5,000 data sets (corresponding to 13 minutes or so). But in the whole period of 30,000 data (corresponding to 1.5 hours), there is still baseline shift.

## Figures and Tables

**Table 1.** Category of sensors according to their power spectra and *pdf*.

*pdf*	Single peak	Double peaks
Power spectra		
Coloured noise	(1) TGS813 etc.	(3) TGS830 etc.
White noise	(2) 3ETO etc.	N/A
